# Round the Hospitals

**Published:** 1921-05-07

**Authors:** 


### ROUND THE HOSPITALS.

Provincial hospitals are beginning to appreciate the importance of arousing interest in their work among the small givers. At the Cheltenham General Hospital there is not only a Ladies' Guild, which divides the town into districts and makes house-to-house calls, but also a Chaplain's Band of Helpers, subscribing not less than a halfpenny a week or 2s. 6d. a year, and making, under the matron's directions, bed-jackets, dressing-gowns, etc., for the patients. At the Warneford and Leamington Hospital a Ladies' Village Association has recently been started, which arranges to canvass the villages for weekly contributions. This has already proved very successful. In our experience nothing stimulates interest in hospital work so much as being encouraged to help in the way of needlework, but this requires good organisation.

Salaries at St. George's Hospital are now as follows: Home sister, £70 to £100; night sister and sister-tutor, £80 to £100; ward sisters, £60 to £90; and by the addition to the staff of twenty nurses the hours on duty have been considerably reduced. Out of the twenty-one staff nurses who completed their training last year, eight have gone to nursing homes, three to private nursing, two are temporary sisters at St. George's, two are sisters at other hospitals, three are taking the C.M.B. certificate, and three have gone home. A system of payments from patients, varying from 10s. 6d. to £3 3s. a week, was inaugurated on September 1, and brought in nearly £2,000 by the end of the year. It has worked smoothly, and does not debar the hospital from admitting really necessitous cases free, as heretofore. The cost of maintenance has more than doubled since 1913, and now stands at £85,626.

The Leeds Hospital for Women and Children has been in such great straits for lack of nurses that for three months it was necessary to close half the wards. A comprehensive scheme for improving the conditions under which the nurses work was drawn up last year, but it was found impossible to put it into practice owing to the scarcity of candidates. Needless to say, the work has pressed very heavily indeed on the diminished staff, who have carried it through, nevertheless, in a spirit of true devotion. Things are now easier, and the wards are being reopened one by one, but even now probationers are hard to attract. Have the improved conditions of work come into force yet? We notice in this connection that the salary offered to a fully-trained staff nurse at this hospital is only £50. The College of Nursing scale for staff nurses is £60 to £70. Small wonder then that this hospital's advertisement for a staff nurse is be seen almost week by week.

Princess Mary has accepted the presidency of the "Not Forgotten" Association, founded by Miss Marta Cunningham, for extending hospitality and distributing gifts to the wounded soldiers still under treatment in London and the provinces. There are 2,000 men in hospital in London and over 8,000 in the region, besides some 64,000 out-patients. A steady series of invitations is aimed at, indoor and out-of-door entertainments, drives, etc. Visitors who will go to the hospitals, make friends with the men, and help to cheer their long leisure are greatly needed. None, we feel sure, would be more warmly welcomed than some of the sisters who followed their fortunes and shared their trials in the war. Perhaps some of our readers who have laid aside their active nursing duties could spare time to visit regularly some of the bed-ridden to whom the country owes so much. Miss Marta Cunningham's address is 86 Ladbroke Road, W. 11.

Members of the College of Nursing who desire to take a course of graduate training to fit them for an administrative or a teaching post should write at once to the Secretary at 7 Henrietta Street, W. 1, for particulars of the sister-tutor studentships which, as will be seen from our advertisement columns, are now being offered. There are five in all, two by the Council, two "Cowdray" studentships for members trained in Aberdeenshire and Sussex respectively, and one by the London Centre, the candidate to be preferably a member of the London Centre. The studentships are of the value of £135 each, tenable at King's College for Women. A qualifying examination has to be undertaken by the candidate, and applications must be received by the Secretary of the College of Nursing before May 28.

There seems some probability that a war memorial to nurses on a national scale may shortly take shape. Up to the present nurses' memorials have been confined to commemorating the services of members of individual institutions and organisations, though the tablet in the Chapel of Kensington Infirmary (which was illustrated in our issue of January 15) was erected as a general memorial to all nurses. Scottish nurses of the Q.A.I.M.N.S. and T.F.N.S. are commemorated by a mural monument shortly to be placed in St. Giles's Cathedral, Edinburgh, and tablets have been placed in St. Asaph's Cathedral, North Wales, and in the

**ROUND THE HOSPITALS**—(*continued*).

Garrison Church, Dublin, in memory of Welsh and Irish nurses. It is suggested that a nurses' memorial should be placed in the Kitchener Memorial Chapel at St. Paul's, and the National Memorial Fund is understood to approve this plan. A Roll of Honour in alabaster will shortly be placed in the Memorial Chapel of Queen Alexandra Military Hospital, Millbank, on which the names of English nurses will be inscribed.

THE Club presented to Aberdeen by Lady Cowdray for nurses and other professional women is to be opened this month. The building is close to Duthie Park, is admirably fitted for its purpose, and has been handsomely equipped. It will be called the Cowdray Club, and was a gift to the College of Nursing Centre in Aberdeen, though with their cordial assent it is to be used by no means exclusively for their benefit.

THE inquest held at Torquay on Daisy Mary Stephen, aged thirty-five, a nurse, who died from taking morphia, reveals yet another case of mental breakdown due to hardships encountered in the war. Miss Stephens did distinguished service in Serbia, was taken prisoner at the fall of Belgrade, and, though eventually released, returned again to Serbia, where she contracted appendicitis. The effects of two operations produced pain and sleeplessness, which ended with the fatal self-administration of the drug. Dr. Stubb, under whom she has recently been working, testified that he had never known a better nurse, and very few as good.

MISS POLLARD and Miss Pearson have been elected by the Council of the Midwives Institute as their representatives on the Central Midwives Board. Miss Pollard, who is a fully trained nurse, is an inspector of midwives and has a wide knowledge of the training of pupils and Poor-Law midwifery. Miss Pearson has had experience in the organisation of the practising midwife, to whose economic position and problems she has given considerable attention.

THE editor of the College of Nursing *Bulletin* has an interesting article in the *Daily Chronicle* on the reasons why nursing is unpopular. We cannot agree with her that one of the chief reasons why nurses are underpaid is the insolvent condition of voluntary hospitals. Underpayment was rife long before the hospitals got into difficulties, and their rates of pay and conditions compare very favourably indeed with those offered in rate-supported institutions.

A MOVEMENT is on foot under Dr. D. Arthur Hughes, M.O.H. for the County of Carmarthen, to establish a County Nursing Association, in distinction from the South Wales Nursing Association. Dr. Hughes is in favour of establishing an institution for training nurses in the county in order to raise the present low standard. The aim is to enlist fifty fully-trained hospital nurses to take the place of the village nurses. It is a bold scheme.

